# Simultaneous Determination of Five Iridoids of *Picrorhiza scrophulariiflora* in Rat Plasma Using UHPLC-ESI-MS/MS

**DOI:** 10.3390/molecules28155925

**Published:** 2023-08-07

**Authors:** Zhibin Wang, Xuepeng Shi, Shuang Jiang, Jiahui Sun, Gilwa Borjigin, Qi Li, Yuanqiu Mu, Chunjuan Yang, Zhenyue Wang, Haixue Kuang

**Affiliations:** 1 Key Laboratory of Basic and Application Research of Beiyao, Ministry of Education, Heilongjiang University of Chinese Medicine, Harbin 150040, China; wzbmailbox@126.com (Z.W.); wangzhen_yue@163.com (Z.W.); 2 Department of Pharmaceutical Analysis and Analytical Chemistry, College of Pharmacy, Harbin Medical University, No. 157 Baojian Road, Nangang District, Harbin 150081, China; sxp08031026@163.com (X.S.); jsjiang111@163.com (S.J.); sunjiahuijhs@163.com (J.S.); borjigingilwa@163.com (G.B.); meshilq@outlook.com (Q.L.); myqj135790@163.com (Y.M.); chunjuanyang@126.com (C.Y.)

**Keywords:** UHPLC-ESI-MS/MS, *Picrorhiza scrophulariiflora*, iridoids, rat, pharmacokinetics

## Abstract

In this study, we developed an ultra-performance liquid chromatography-electrospray tandem quadrupole mass spectrometry (UHPLC-ESI-MS/MS) method to simultaneously determine Picroside-I, Picroside-II, Picroside-III, minecoside, and sweroside in rat plasma. The chromatographic column was an ACQUITY UHPLC^®^ BEH Amide Column (2.1 × 100 mm, 1.7 µm; Waters, MA, USA), column temperature 40 °C. The mobile phase was 0.1% formic acid aqueous solution–0.1% formic acid acetonitrile solution. The flow rate was 0.4 mL/min. Multiple reaction monitoring (MRM) and negative ion modes were adopted. The results showed that the calibration curves of five compounds in plasma showed good linearity (*r* > 0.9911) over the studied dose range. The lower limits of quantification (LLOQ) for Picroside-I, Picroside-II, Picroside-III, minecoside, and sweroside were 6.876, 5.193, 5.040, 1.260, and 4.527 ng/mL, respectively. The intra-day and inter-day precision were <15%. The matrix effects ranged from 95.77 to 101.9%. The *T*_max_ were 1.1 ± 0.2, 1.1 ± 0.1, 0.8 ± 0.1, 1.0 ± 0.2, and 2.1 ± 0.1 h. This study will be useful in understanding the behavior of drugs in the body and the body’s effect on drugs. It also offers theoretical underpinnings and highlights the importance of clinical applications and creating novel drugs.

## 1. Introduction

**Figure 1:**
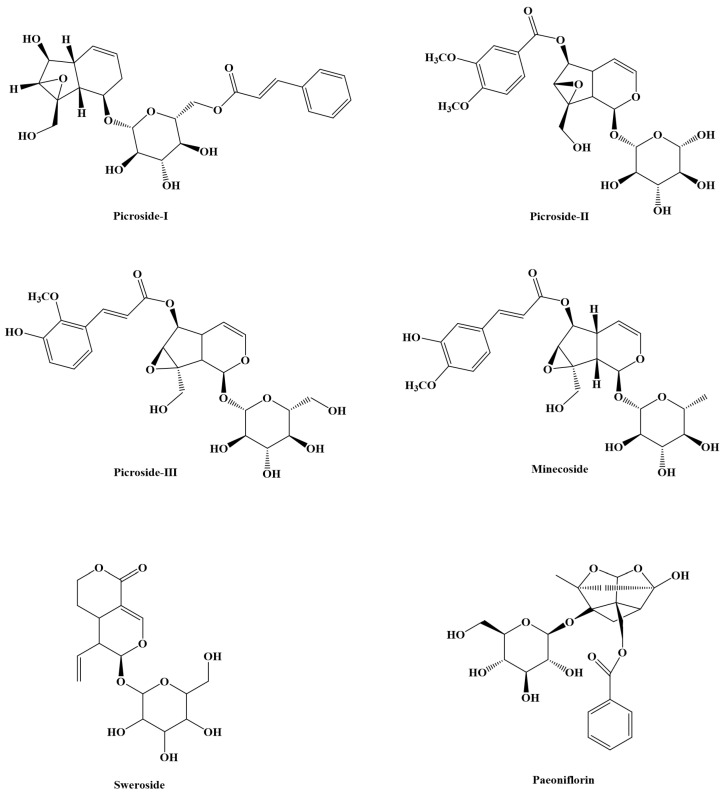
The chemical structures of Picroside-I, Picroside-II, Picroside-III, minecoside, and sweroside.

*Picrorhiza scrophulariiflora* (PS) is the dried rhizome of the *Scrophulariaceae* plant *Picrorhiza scrophulariiflora Pennell*, which was first published in *Sheng Nong’s herbal classic*. In traditional Chinese medicine (TCM), the dry roots and rhizomes can abate vacuity heat, chancroid fever, and damp-clearing heat. It is usually used for bone-steaming hot flashes (chronic fatigue syndrome), infantile chancre fever, damp-heat diarrhea (vascular occlusion, intestinal dysfunction), jaundice, and urine redness (called neonatal hyperbilirubinemia in modern medicine), sores, etc. It mainly contains iridoids, phenolic glycosides, phenol glycosides, and other compounds. Of these, iridoids are numerous with the most variety. They are the primary active ingredient in PS [1], Picroside-I [2], and Picroside-II [3], which are index components specified in the *Chinese Pharmacopoeia.* To evaluate the quality of this medicinal material, Picroside-I, Picroside II, and Picroside III [4] are the three main active ingredients in PS, which have shown significant effects on the liver and gallbladder in previous pharmacological studies. They also have various pharmacological effects such as antibacterial, anti-inflammatory, antioxidant, anti-asthma, anti-myocardial ischemia, anti-hepatitis-B virus, tumor inhibition, and multi-drug resistance [5,6,7,8,9,10,11]. Furthermore, minecoside [12] and sweroside [13] in PS have garnered recent interest (Figure 1). Minecoside has hyaluronidase inhibitory activity [14]. Minecoside also inhibits the expression of CXCR4 in breast cancer cells, and its inhibitory effect on CXCR4 expression is not limited to specific cancer cells [15]. Sweroside has a wide range of pharmacological and biological effects, including osteoblast generation [16,17] and anti-inflammatory effects [18]. Sweroside can reduce aconitine’s toxicity to myoblasts of the cardiac cell line H9c2 [19]. Sweroside also improves pro-inflammatory responses and bile acid levels in mice to treat bile stasis-induced liver injury caused by isothiocyanate on naphthalene [20].

Although these five iridoids’ pharmacological activity, mode of action, and clinical applications have been extensively documented, their pharmacokinetic characteristics are still unclear. Only a few papers have provided adequate pharmacokinetic parameters for Picroside-I, II, III [21], and sweroside [22,23]. The pharmacokinetics of minecoside have not been reported before. TCM frequently combines a number of different components. Several active substances may interact during the metabolic process. Therefore, pharmacokinetically studying the combined use of these five compounds will provide valuable information. At the same time, studying five iridoid compounds may be more reasonable and effective than studying single components. Therefore, a method to simultaneously determine these five compounds is being sought.

Pharmacokinetics mainly studies the dynamic changes of drug disposal in the body, including the absorption, distribution, biochemical conversion (or metabolism), and excretion processes of drugs within the body, especially changes in blood drug concentration over time. Demands for the pharmacokinetic qualities of pharmaceuticals are increasing along with drug research’s advancement and improvements in human health. It is important to consider a drug’s pharmacokinetic qualities as well as its potency and side effects when assessing its potential uses. TLC (thin-layer chromatography) [24], HPLC-UV [25], HPLC-ELSD (evaporative light scattering detection) [26], LC-MS/MS [27], and several high-performance liquid chromatography methods with UV detection have been used to analyze iridoid glycosides.

In recent years, a range of methods have been used to investigate iridoids and understand their pharmacokinetic role in PS for both animals and people. However, we determined the pharmacokinetic behaviors of Picroside-I, Picroside-II, Picroside-III, minecoside, and sweroside in rat plasma simultaneously (Figure 1), which was not reported in any research publications. This article is the first to simultaneously determine the pharmacokinetic behavior of PS extract in rat plasma, providing a reference for the clinical treatment of PS. The usage of LC-MS technology in the field of drug analysis is growing, fully demonstrating how chromatography and mass spectrometry have complementary advantages. Combining high selectivity, sensitivity, and the ability to deliver relative molecular weight and structural information swiftly and efficiently with chromatography’s great separation capabilities for complex materials enhances this method.

In this paper, we propose a UHPLC-ESI-MS/MS method to measure the concentrations of Picroside-I, Picroside-II, Picroside-III, minecoside, and sweroside in rat plasma. We studied these compounds’ pharmacokinetics in rats and provided a scientific basis for the medicinal value of PS and regulating their clinical drug use.

## 2. Results and Discussion

### 2.1. Optimization of Chromatographic and Mass Spectrometric Conditions

Compared to traditional HPLC, the chromatographic column packing used in UHPLC is smaller (1.8 μm), which can significantly improve the separation of peaks and shorten the analysis time of samples, saving the mobile phase. To shorten the analysis time, improve the peak shape, and effectively increase the peak signal response intensity, etc., the mobile phase conditions were optimized as follows: The methanol–water and acetonitrile–water systems were used as the mobile phases, and the mobile phase pH was compared by adding different concentrations of formic acid, acetic acid, and ammonium acetate. Our results showed that adding 0.1% formal acid to the acetonitrile–water system could enhance the signal response intensity of the analyses to be measured, thereby shortening the retention time and obtaining a satisfactory baseline with good selectivity, low background noise, and good peak shape. Therefore, 0.1% formic acid in the mobile phase was selected as the detection condition. The five analytes’ separation, peak shape, response values, and internal standard were optimized by an acetonitrile–0.1% formic acid–water system. In this study, the peaks of the analytes and IS were obtained within 3 min, which greatly shortened the analysis time and saved the use of mobile phase and nitrogen gas.

In this study, MRM multiple reaction monitoring modes were used as the scanning method for quantitatively determining five compounds in PS. In other words, a first mass analyzer (Q1) selected quasi-molecular ions from the analytes and the internal standard as precursor ions (or parent ions). These precursor ions were cleaved by collision with collision gas (N_2_) in the collision cell (Q2) to form fragments for generating product ions (or daughter ions). A third mass analyzer (Q3) was used to select major product ions from the analytes and an internal standard to monitor their chromatographic peak area ratio. We quantified the components being measured. Our results showed that the five analytes and internal standard were more stable in the negative ion mode than in the positive ion mode.

### 2.2. Selection of Internal Standard

The chromatographic and mass spectrometric behaviors and extraction recovery of the internal standard should be close to those of the analytes. Compounds with similar structures or retention behaviors to those of the analytes are often selected as IS in experiments. Paeoniflorin was selected as the internal standard. Our results showed that Paeoniflorin was an ideal internal standard for this experiment because of its good response, peak shape, short retention time, and no interference with other analytes under the established conditions.

### 2.3. Optimization of Plasma Sample Processing Method

Common pretreatment methods used for determining biological samples are liquid–liquid extraction (LLE), solid-phase extraction (SPE), and precipitated protein (PPT). Among the sample pretreatments, the precipitated protein method suits compounds with high polarity, simple operation, and good extraction recovery. In this experiment, we investigated the methanolic protein precipitation and acetonitrile protein precipitation methods. Our results showed that the acetonitrile protein precipitation method had a lower minimum limit of quantification, better peak shape, lower noise level, and better reproducibility. The extraction recoveries for all five analytes were >64.0%. Therefore, we selected the acetonitrile protein precipitation method as the plasma sample processing method.

### 2.4. UHPLC-MS/MS Method Validation

#### 2.4.1. Specificity

**Figure 2:**
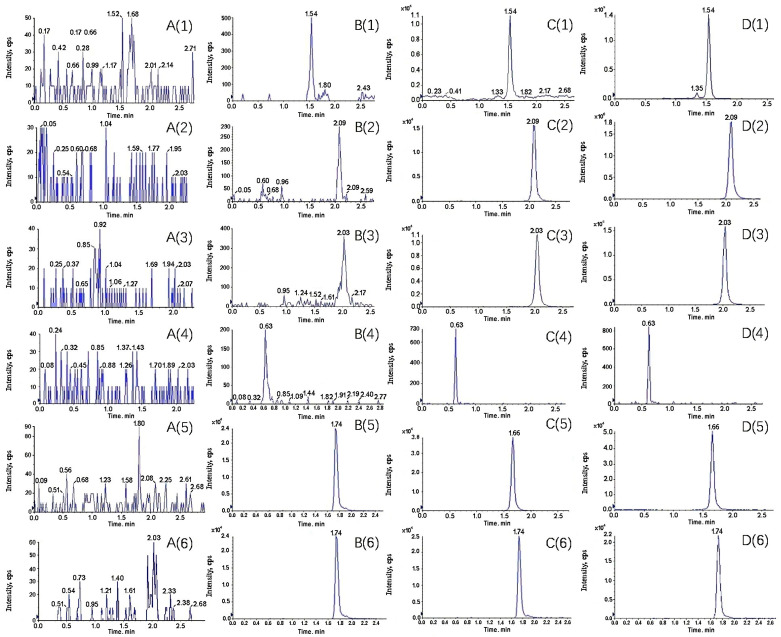
Typical MRM chromatograms of Picroside-I (**1**), Picroside-II (**2**), Picroside-III (**3**), minecoside (**4**), sweroside (**5**), and the internal standard Paeoniflorin (**6**) in plasma samples. (**A**) Blank plasma; (**B**) blank plasma sample with the addition of analyte (LLOQ) and an internal standard; (**C**) blank plasma samples with an analyte (MQC) and internal standard; (**D**) rat plasma samples 1 h after oral administration of PS extract.

**Figure 3:**
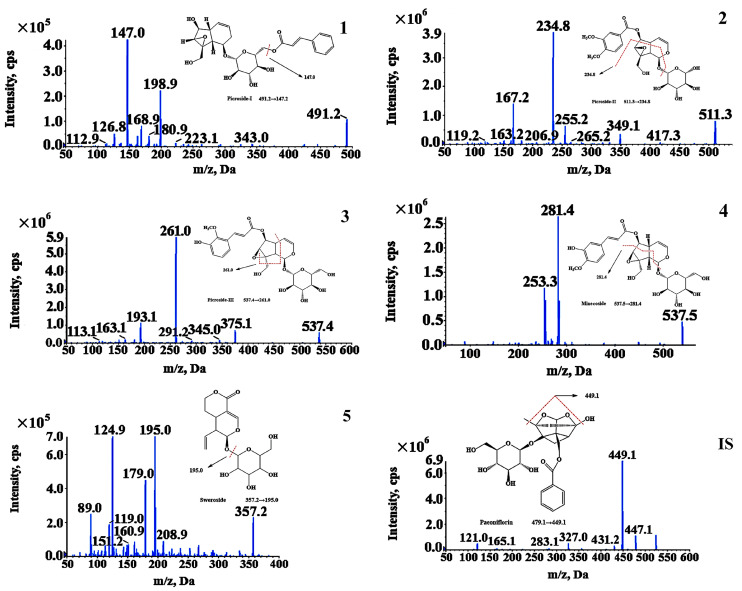
Picroside-I (**1**), Picroside-II (**2**), Picroside-III (**3**), minecoside (**4**), sweroside (**5**) and internal standard (IS) ions.

Figure 2 and Figure 3 shows retention times and representative chromatograms of rat blank plasma, blank plasma mixed with control for the test (LLOQ) and internal standard, blank plasma mixed with control for the test (QC), and plasma samples after 1.0 h of oral administration of PS extract. Picroside-I, II, III, minecoside, sweroside, and paeoniflorin retention times were 1.54, 2.09, 2.03, 0.63, 1.66, and 1.74 min, respectively. The five analytes and IS had good responses and no significant interference within the retention times.

#### 2.4.2. Linearity and Lowest Limit of Quantification (LLOQ)

The linear range of the five analytes was 6.876–764.0 ng/mL for Picroside-I, 5.190–577.0 ng/mL for Picroside-II, 5.040–560.0 ng/mL for Picroside-III, 1.260–140.0 ng/mL for minecoside, and 4.572–503.0 ng/mL for sweroside, respectively. The concentration of the internal standard substance was 527.0 ng/mL. Three samples of each concentration were measured horizontally. Table 1 shows the linear regression equation, linear range and LLOQ of the five compounds. All five analytes showed good linearity (*r* ≥ 0.9911).

#### 2.4.3. Extraction Recovery and Matrix Effect

The extraction recoveries of the five analytes at three QC concentrations were 86.51, 85.12, and 91.34% (Picroside-I); 71.02, 67.11, and 72.53% (Picroside-II); 76.43, 80.47, and 83.84% (Picroside-III); 77.62, 83.16, and 80.45% (minecoside); 82.25, 85.36, and 79.57% (sweroside), respectively. The matrix effect of the five substances under three QC concentrations was 96.17, 101.9, and 98.93% (Picroside-I); 101.7, 99.71, and 97.26% (Picroside-II); 95.77, 99.32, and 101.8% (Picroside-III); 96.84, 97.17, and 97.47% (minecoside); 98.61, 98.65, and 101.5% (sweroside), respectively. The results are shown in Table 2.

#### 2.4.4. Intra-Day and Inter-Day Precision and Accuracy

Table 3 summarizes the intra-day and inter-day precision and accuracy of Picroside-I, -II, -III, minecoside, and sweroside according to four levels of QC samples (*n* = 6) in plasma. All assay values indicated that the accuracy and precision of the method were acceptable for biological sample analysis.

#### 2.4.5. Stability

We investigated the stability of Picroside-I, -II, -III, minecoside, and sweroside in rat plasma under various storage and processing conditions. The data are presented in Table 4. Our results indicated that Picroside-I, -II, -III, minecoside, and sweroside were stable (with ±15% for R.E., RSD < 15%) at the anticipated conditions, including three freeze/thaw cycles at room temperature for 4 h and in a freezer set to −20 °C for 14 days. An autosampler stability test also suggested that Picroside-I, -II, -III, minecoside, and sweroside were stable (with ±15% for R.E., RSD < 15%) in the mobile phase at 4 °C for at least 24 h.

### 2.5. Pharmacokinetic Characteristics

**Figure 4:**
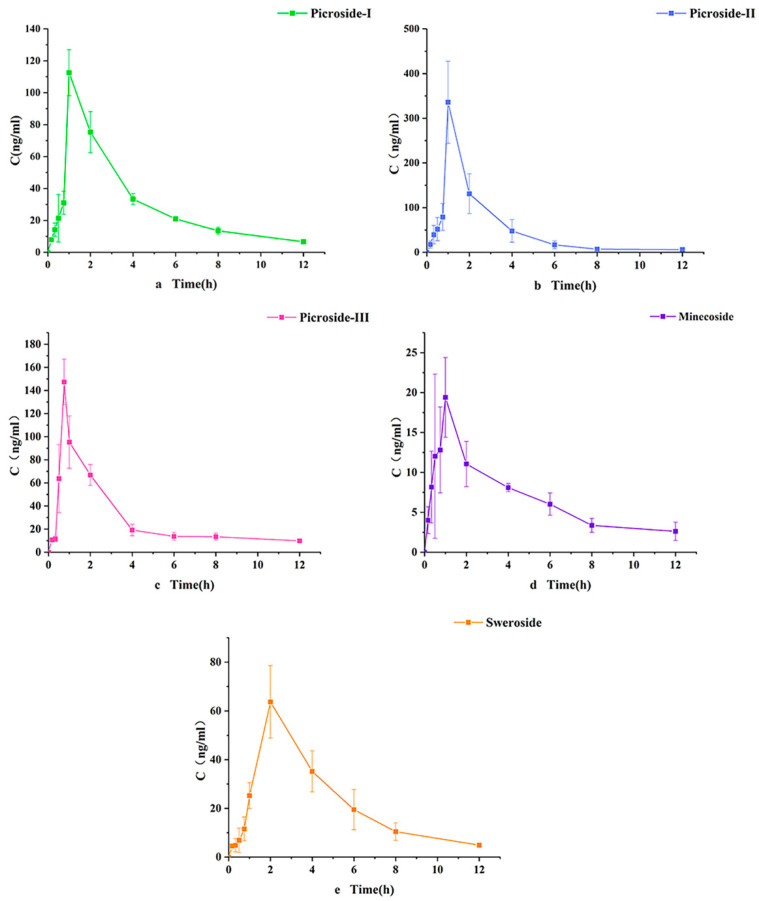
Mean blood time profiles of the five analytes in rat plasma after oral administration of Picrorhiza extract (*n* = 6). (**a**) Picroside-I, (**b**) Picroside-II, (**c**) Picroside-III, (**d**) minecoside. and (**e**) sweroside.

In this study, we developed an efficient and reliable UHPLC-ESI-MS-MS analytical method to simultaneously determine five iridoid components in SD rats’ plasma after oral administration of PS. In this study, the systemic exposure of Picroside-II had the largest (AUC_0–t_: 603.9 ± 239.4 h ng/mL), similar to previous studies [21]. Table 5 shows the AUC_0–t_ of the five compounds. The time to reach the maximum drug concentrations (*T*_max_) for Picroside-I, Picroside-II, Picroside-III, minecoside, and sweroside were 1.1 ± 0.2, 1.1 ± 0.1, 0.8 ± 0.1, 1.0 ± 0.2, and 2.1 ± 0.1 h, respectively. The t_1/2_ were 2.4 ± 0.3, 2.3 ± 1.5, 17.5 ± 3.1, 5.0 ± 2.3, 1.2 ± 0.3 h, and MRT_0–t_ were 3.6 ± 0.2, 2.3 ± 0.5, 3.4 ± 0.1, 4.1 ± 0.7, and 3.9 ± 0.3 h, respectively. These findings indicate that iridoid compounds have a short retention time in the body, are rapidly eliminated, and are less likely to accumulate, resulting in low bioavailability. Since these five iridoids have a lactone ring structure, they may encounter gastric acid and partial intestinal acid environment instability after administration, resulting in low absolute bioavailability [28]. The t_1/2_ of the five iridoids is somewhat different from previous studies [20,21,22], perhaps due to different doses and drug extraction methods. In addition, unlike other studies, we administered PS extracts rather than monomer compounds to experimental animals. Different drug types and complex components in TCM may be the reason for differences in the t_1/2_ of iridoids. The *C*_max_ of the five iridoids were 114.2 ± 16.1, 335.9 ± 92.3, 148.2 ± 21.6, 21.1 ± 7.8, and 63.7 ± 17.6 ng/mL, respectively. Unlike previous experimental research data, the *C*_max_ is different because of content differences in these compounds in PS. PS extracts are more conducive to clinically treating TCM than directly administering monomer compounds as TCM is usually administered as a mixture to patients. This paper reports the pharmacokinetics of minecoside for the first time. The *C*_max_ values of minecoside and sweroside are relatively low compared to the other three compounds, possibly due to their low content amounts in PS. This study provides important information for evaluating the pharmacokinetics of TCM PS and promotes its efficacy in clinical treatment research. Table 5 shows the final calculated pharmacokinetic parameters. The mean blood concentration–time curve is plotted in Figure 4.

## 3. Materials and Methods

### 3.1. Chemicals and Reagents

Picroside-I, Picroside-II, Picroside-III, minecoside, and sweroside were isolated, determined by HPLC, and calculated as ≥98% by the normalization method, which can be used as content determination. The internal standard Paeoniflorin (≥99%) was purchased from Nanjing Jingzhu Bioscience Co (Nanjing, China). PS was purchased from Hui ren dang and identified as the dried rhizome of *Picrorhiza scrophulariiflora Pennell* by Prof. Zhenyue Wang of the Department of Chinese Medicine Resources, Heilongjiang University of TCM. Methanol and acetonitrile were purchased from Thermo Fisher Scientific (Waltham, MA, USA). Formic acid was purchased from Dikma (Beijing, China), ether was purchased from Tianjin Bodi Chemical Co. (Tianjin, China), and heparin was purchased from Shanghai First Biochemical Pharmaceutical Co. (Shanghai, China). Distilled water was purchased from A.S. Watson Group (Hong Kong) Co. (Hong Kong, China). All other chemicals were of analytical grade.

### 3.2. Preparation of Standard Solutions

We accurately weighed the reference substances of Picroside-I, -II, -III, minecoside, sweroside, and Paeoniflorin and prepared a mixed solution with methanol. The five substance concentrations in the mixed solution were 1.53, 1.15, 1.12, 0.56, 1.00, and 1.05 mg/mL, respectively. This solution was used as a reference stock solution and stored at 4 °C. We diluted the reference stock solution with methanol, and the Picroside-I concentrations in the diluted standard solution were 6.876, 13.75, 34.38, 57.30, 114.6, 191.0, 382.0, and 764.0 ng/mL, respectively. The Picroside-II concentrations were 5.193, 10.39, 25.97, 43.28, 86.55, 144.3, 288.5, and 577.0 ng/mL, respectively. The Picroside-III concentrations were 5.040, 10.08, 25.20, 42.00, 84.00, 140.0, 280.0, and 560.0 ng/mL, respectively. The sweroside concentrations were 4.527, 9.054, 22.64, 37.73, 75.45, 125.8, 251.5, and 503.0 ng/mL, respectively. The minecoside concentrations were 1.260, 2.520, 6.300, 10.50, 21.00, 35.00, 70.00, and 140.0 ng/mL series standard solution, respectively. We diluted the internal standard paeoniflorin to 527.0 ng/mL and refrigerate it at 4 °C. The same method was used to create three quality control samples (QC) of high (HQC), medium (MQC), and low (LQC) concentrations: 13.75, 382.0, and 573.0 ng/mL for Picroside-I; 10.39, 288.5, and 432.8 ng/mL for Picroside-II; 10.08, 280.0 and 420.0 ng/mL for Picroside-III; 9.054, 251.5, and 377.2 ng/mL for sweroside; and 2.520, 70.00, and 105.0 ng/mL for minecoside.

### 3.3. Processing of Plasma Samples

We obtained 100 µL of the standard solution in “A” and added it to a 2 mL glass tube. We then added 100 µL of blank plasma, 20 µL of internal standard solution, and 400 µL of acetonitrile to precipitate the protein. The mixture was vortexed for 3 min, centrifuged at 3500 rpm for 10 min, and the supernatant was transferred to a clean 10 mL glass tube and dried under a nitrogen flow at 30 °C. The residue was resolubilized with 100 µL of the initial mobile phase, sonicated for 1 min, vortexed for 3 min, and then transferred to a 1.5 mL centrifuge tube for 10 min at 12,000 rpm at 4 °C. We injected 2 µL of the supernatant into the UHPLC-MS/MS system for sample analysis.

### 3.4. Instrumentation and LC–MS/MS Analytical Conditions

The UHPLC–MS/MS system consisted of a Waters UHPLC system and AB SCIEX 4000 QTRAP triple quadrupole linear ion trap tandem mass spectrometer with an electrospray ionization (ESI) interface source. Chromatographic separation was performed with an ACQUITY UHPLC^®^ BEH Amide Column (2.1 × 100 mm, 1.7 µm). Mobile phases were 0.1% of formic acid in water (A) and 0.1% of formic acid in acetonitrile (B), isocratic elution: 0–3 min, A:B = 8:92 (*v*/*v*), flow rate of 0.4 mL/min, and column temperature of 40 °C. The injection volume was 2 µL.

The operation MS parameters were as follows: detection mode—negative ion mode, scan mode—multiple reaction monitoring mode (MRM), the dwell time was 200 ms, source injection voltage was −4500 V, ion source temperature was 450 °C, nebulizing gas was 55 psi, and heating gas was 55 psi. The detection ion pairs for the five compounds and IS were Picroside-I *m/z* 491.2/147.0, Picroside-II *m/z* 511.3/234.8, Picroside-III *m/z* 537.4/261.0, minecoside *m/z* 537.5/281.4, sweroside *m/z* 403.2/195.0, and IS *m/z* 525.2/449.1. Table 6 shows the secondary full scan mass spectra, declustering potential (DP), and collision energy (CE) of the analytes and internal standard.

### 3.5. Validation of Analytical Methods for Plasma Samples

#### 3.5.1. Specificity

The specificity of the method refers to determining a component that can be accurately measured when other interfering substances are present. Samples were obtained and analyzed according to the sample preparation method described in Section 3.3. We also compared chromatograms of the blank plasma, the blank plasma mixed with the control (LLOQ) and IS of the substance being measured, the blank plasma mixed with the control (MQC) and IS of the substance being measured, and the plasma samples after 1.0 h of oral administration of PS extract. The plasma samples were chromatographed to examine whether endogenous substances in the plasma interfered with the test and IS. All validation programs referred to the US Food and Drug Administration (FDA) guidelines for biological method validation, which can be obtained online (https://www.fda.gov/media/162903/download, accessed on 26 July 2023).

#### 3.5.2. Linearity and Lowest Limit of Quantification (LLOQ)

We assigned the concentrations (C) of Picroside-I, Picroside-II, Picroside-III, minecoside, and sweroside in plasma as the abscissa, and the ratio of the analyte’s peak area to the internal standard (Y) as the ordinate. We determined seven different concentration levels and used the weighted least square method (W = 1/χ^2^) for regression calculation to obtain the regression equation and calculate the linear regression coefficient r. The lower limit of quantification (LLOQ) was the lowest point on the standard curve. The LLOQ was defined as the lowest concentration of the analyte whose signal-to-noise (S/N) ratio was >10:1.

#### 3.5.3. Extraction Recovery and Matrix Effects

The extraction recovery rate, which is typically expressed in percent, is the ratio of the response value of the substance recovered from the biological sample matrix to the response value produced by the pure solution with the QC sample concentration to the biological matrix sample, which is used as a control after extraction. (1) Obtain 100 µL of blank plasma from SD rats, and add QC samples at low, medium, and high concentrations of 100 µL. According to the method described in Section 3.3, prepare QC samples with low, medium, and high concentrations of five compounds, namely Picroside-I, Picroside-II, Picroside-III, minecoside, and sweroside. Obtain 2 µL for injection analysis. Prepare six samples in parallel for each sample. Record the chromatogram to obtain the analytes’ ratio of chromatographic peak areas (A1) to the internal standard. (2) Obtain 100 µL of rat blank plasma after treatment according to the preparation method described in Section 3.3. Add QC samples at low, medium, and high concentrations of 100 µL before nitrogen blowing. Blow them dry together and redissolve them in the mobile phase. Obtain 2 µL for injection and analysis, prepare six samples in parallel for each sample, record the chromatogram, and obtain the analytes’ ratio of chromatographic peak areas (A2) to the internal standard. The extraction recovery rate is A1/A2 × 100%.

#### 3.5.4. Intra-Day and Inter-Day Precision and Accuracy

We obtained 100 µL of blank plasma from SD rats and the samples were processed according to the method described in Section 3.3. Four concentrations of QC samples were prepared, namely HQC, MQC, LQC, and LLOQ. For each concentration, six samples were analyzed for three consecutive days, and the QC sample concentrations were calculated by an accompanying standard curve on the same day. We also obtained the inter-day and intra-day precision RSD% and accuracy RE%. The criteria for data acceptability included accuracy within ±15% relative error (RE) from the nominal values and a precision of ±15% (RSD). The accuracy of the average value of LLOQ samples compared to the indicated value should not exceed ±20%, and the precision should not exceed 20%.

#### 3.5.5. Stability

Stability refers to the chemical stability of the sample being tested in a given medium under certain external conditions. We obtained 100 µL of rat blank plasma and prepared low, medium, and high concentrations of the QC sample standard solutions, Picroside-I, Picroside-II, Picroside-III, minecoside, and sweroside, according to the method described in Section 3.3. As simulated biological samples, the following conditions were investigated: (1) Short-term stability (QC samples were left at room temperature for 4 h, which exceeded the sample preparation time); (2) long-term stability (QC samples were left at −20 °C for two weeks); (3) freeze-thaw stability (three freeze-thaw cycles at −20 °C/room temperature) and post-preparation stability (QC samples were left in a 4 °C auto sampling chamber for 24 h).

### 3.6. Pharmacokinetic and Data Analysis

#### 3.6.1. Animals

Six adult male SD (Sprague Dawley) rats (220–250 g) were purchased from the Animal Experiment Center of Heilongjiang University of Chinese Medicine. The Animal Ethics Committee of Harbin Medical University approved the experimental protocol, which conformed to the principles for the Care and Use of Laboratory Animals. The experimental animals were fed under the following conditions: room temperature (20–25 °C), relative humidity (40–60%), 12 h of alternating day and night lighting, 12 h of fasting before the experiment, and free access to water. We calculated the dosage of SD rats using the standardized formula for body surface area (BSA). According to the *Chinese Pharmacopoeia* [2], the dosage of PS raw medicine for human use is 10 g. The dosage for rats is 0.9 g/kg.

#### 3.6.2. Preparation of PS Gavage Solution

The dried PS powder weighed 200 g and was extracted with eight times the amount of 75% ethanol at reflux three times for 2 h each time. The extracts were combined, filtered, distilled under reduced pressure until the alcoholic taste disappeared, and lyophilized with water. In our previous laboratory experiments, we used HPLC to determine the content of five analytes, namely Picroside-I (15.68 mg/g), Picroside-II (103.64 mg/g), Picroside-III (64.08 mg/g), minecoside (2.02 mg/g), and sweroside (12.28 mg/g). After freeze-drying, we dissolved the solid in a 5% sodium carboxymethyl cellulose solution and prepared a medicinal extract solution with a concentration of 0.3 g/mL.

#### 3.6.3. Dosing Regimen and Sample Collection and Processing

A dose of 1 g/kg of body weight was administered to rats according to the pharmacopoeia and previous content results for the five analytes. Rat tail vein blood samples were collected in heparinized tubes before and 0.167, 0.333, 0.5, 0.75, 1, 2, 4, 6, 8, and 12 h after administration and centrifuged at 3500 rpm for 10 min. We obtained 100 µL of the upper layer of plasma and stored it at −20 °C for measurement.

#### 3.6.4. Plasma Sample Determination

The plasma samples were treated according to the method described in Section 3.3. The plasma samples were determined according to the chromatographic conditions described in Section 3.4 and chromatograms were recorded. The measured peak area ratios of the substances being measured and the IS were substituted into the accompanying standard curves on the same day. We also calculated the blood concentrations of Picroside-I (15.68 mg/g), Picroside-II (103.64 mg/g), Picroside-III (64.08 mg/g), minecoside (2.02 mg/g), and sweroside (12.28 mg/g), respectively. We determined the QC samples at the same time, allowing a maximum of two different concentrations of QC samples to exceed the theoretical value of ±15% of the RE% value.

#### 3.6.5. Calculation of Pharmacokinetic Parameters

The peak areas of Picroside-I, Picroside-II, Picroside-III, minecoside, sweroside, and IS in each sample were recorded. We calculated and substituted their ratios into the standard curve equation to obtain corresponding concentrations for the five substances being measured in the plasma of SD rats. The blood concentration data of each rat were processed separately to calculate the relevant pharmacokinetic parameters, and the mean and standard deviation of the parameters were further calculated. We calculated and plotted pharmacokinetic parameters including concentration–time curves (AUC_0–t_, AUC_0–∞_), average residence time (MRT), maximum plasma concentration (*C*_max_), time to reach the maximum concentration (*T*_max_), and half-time (*t*_1/2_) using pharmacokinetic software (DAS 2.0) with a non-atrial chamber model for blood concentration–time curves. All parameters were expressed as mean ± SD.

## 4. Conclusions

We successfully developed and applied a simple, rapid, sensitive, and validated UHPLC-ESI-MS/MS method for the simultaneous determination of Picroside-I, Picroside-II, Picroside-III, minecoside, and sweroside in rat plasma and tissue to the pharmacokinetic study of five iridoid components. As far as we know, this is the first study to report the pharmacokinetics of minecoside and five iridoid components simultaneously. Our study is conducive to a more in-depth study of the absorption process of *Picrorhiza scrophulariiflora* extract in vivo. It is also conducive to the application of this TCM in clinical treatment.

## Figures and Tables

**Table 1:** Regression equations, linear range correlation coefficients, and lowest limit of quantification (LLOQ) for the five analytes.

ID	Regression Equation	Linear Range (ng/mL)	*r*	LLOQ (ng/mL)
Picroside-I	*Y* = 1.32 × 10^−3^*X* + 2.7 × 10^−3^	6.876–764.0	0.9973	6.876
Picroside-II	*Y* = 8.51 × 10^−3^*X* + 6.2 × 10^−2^	5.193–577.0	0.9983	5.193
Picroside-III	*Y* = 0.82 × 10^−3^*X* + 0.6 × 10^−3^	5.040–560.0	0.9954	5.040
Minecoside	*Y* = 5.32 × 10^−3^*X* − 1.3 × 10^−1^	1.260–140.0	0.9911	1.260
Sweroside	*Y* = 2.20 × 10^−2^*X* + 0.4 × 10^−2^	4.527–503.0	0.9989	4.527

**Table 2:** SD Extraction recovery and matrix effect of Picroside-I, -II, -III, minecoside, and sweroside in rat plasma (*n* = 6).

Compound	QC (ng/mL)	Recovery		Matrix Effect	
		Mean ± SD (%)	RSD (%)	Mean ± SD (%)	RSD (%)
Picroside-I	13.75	86.51 ± 4.2	4.9	96.17 ± 2.6	2.7
	382.0	85.12 ± 3.6	4.2	101.9 ± 5.4	5.3
	573.0	91.34 ± 2.3	2.5	98.93 ± 6.4	6.5
Picroside-II	10.39	71.02 ± 8.2	11.5	101.7 ± 7.6	7.5
	288.5	67.11 ± 4.0	6.0	99.71 ± 7.2	7.2
	432.8	72.53 ± 4.6	6.3	97.26 ± 5.1	5.2
Picroside-III	10.08	76.43 ± 6.1	8.0	95.77 ± 3.7	3.9
	280.0	80.47 ± 3.3	4.1	99.32 ± 2.9	2.9
	420.0	83.84 ± 2.5	3.0	101.8 ± 4.8	4.7
Minecoside	2.520	77.62 ± 8.9	11.4	96.84 ± 5.4	5.6
	70.00	83.16 ± 6.1	7.3	97.17 ± 2.6	2.7
	105.00	80.45 ± 3.7	4.6	97.47 ± 5.1	5.2
Sweroside	9.054	82.25 ± 6.8	8.3	98.61 ± 7.5	7.6
	251.5	85.36 ± 5.6	6.6	98.65 ± 6.4	6.5
	377.2	79.57 ± 2.0	2.5	101.5 ± 3.7	3.6

**Table 3:** Results of precision and accuracy tests for five analytes in blank plasma samples from SD rats (*n* = 6).

ID	QC (ng/mL)	Inter-Day		Intra-Day	
		RSD (%)	RE (%)	RSD (%)	RE (%)
Picroside-I	6.876	5.5	1.3	7.7	6.1
	13.75	6.8	1.2	7.9	5.8
	382.0	8.9	1.1	6.9	4.1
	573.0	5.6	2.6	6.6	3.1
Picroside-II	5.193	5.7	12.9	9.7	6.3
	10.39	4.8	−12.1	9.4	−4.3
	288.5	8.5	13.2	10.1	7.1
	432.8	6.1	8.7	4.2	2.9
Picroside-III	5.040	6.2	11.5	8.1	6.8
	10.08	5.8	−11.3	7.6	−3.4
	280.0	7.5	6.5	5.0	6.6
	420.0	5.9	2.0	5.8	6.3
Minecoside	1.260	10.2	12.8	7.0	12.9
	2.520	9.7	−12.3	4.1	13.2
	70.00	6.5	5.6	6.5	8.7
	105.00	11.1	2.2	6.7	−10.3
Sweroside	4.527	9.1	10.8	9.4	10.7
	9.054	7.6	11.2	7.8	6.3
	251.5	9.0	−4.8	9.1	3.5
	377.2	6.8	8.8	8.8	11.3

**Table 4:** Stability of the five analytes in rat plasma samples (*n* = 6).

ID	QC (ng/mL)	Short-Term Stability		Stability after Preparation		Freeze-Thaw		Long-Term Stability	
		RSD (%)	RE (%)	RSD (%)	RE (%)	RSD (%)	RE (%)	RSD (%)	RE (%)
Picroside-I	13.75	5.1	8.93	5.8	6.80	7.1	6.43	10.9	5.60
	382.0	2.9	3.83	6.8	9.73	5.0	−2.74	8.1	−5.69
	573.0	2.9	6.58	4.7	7.02	5.3	−1.94	7.7	3.59
Picroside-II	10.39	4.4	−4.99	4.4	−3.26	4.2	7.08	7.6	8.69
	288.5	3.6	8.53	5.0	6.66	5.9	−7.21	6.6	1.37
	432.8	2.7	4.47	2.8	−6.58	4.3	−1.47	5.0	−2.19
Picroside-III	10.08	4.5	−2.91	3.0	9.08	6.3	2.75	11.1	5.66
	280.0	3.2	6.82	5.5	7.95	5.1	−5.33	6.3	−1.92
	420.0	3.1	4.51	3.4	−4.87	3.5	−1.64	4.2	−4.99
Minecoside	2.520	4.5	3.33	3.3	6.38	11.1	−1.90	4.5	3.31
	70.00	3.2	5.72	5.7	5.13	6.3	7.16	2.2	5.70
	105.00	3.1	3.40	3.4	3.56	4.2	−6.28	2.1	3.41
Sweroside	9.054	1.9	3.68	4.7	9.17	5.3	−1.94	7.7	3.59
	251.5	4.3	−5.09	2.4	4.06	4.2	7.08	7.6	8.69
	377.2	1.6	9.62	5.0	6.66	5.9	−7.21	6.6	1.37

**Table 5:** Pharmacokinetic parameters of each analyte after oral administration of the PS extract (*n* = 6).

ID	*C*_max_ (ng/mL)	*T*_max_ (h)	t_1/2_ (h)	AUC_0–t_ (ng h/mL)	AUC_0–∞_ (ng h/mL)	MRT_0–t_ (h)	Cl (L/h/kg)	Vd (L/kg)
Picroside-I	114.2 ± 16.1	1.1 ± 0.2	2.4 ± 0.3	360.9 ± 25.6	381.8 ± 29.4	3.6 ± 0.2	36.43 ± 17.436	186.7 ± 17.436
Picroside-II	335.9 ± 92.3	1.1 ± 0.1	2.3 ± 1.5	603.9 ± 239.4	610.8 ± 246.1	2.3 ± 0.5	172.40 ± 59.96	796 ± 503.5
Picroside-III	148.2 ± 21.6	0.8 ± 0.1	17.5 ± 3.1	339.4 ± 56.1	597.6 ± 198.1	3.4 ± 0.1	90.07 ± 34.33	2747 ± 1642
Minecoside	21.1 ± 7.8	1.0 ± 0.2	5.0 ± 2.3	78.9 ± 10.2	93.5 ± 22.6	4.1 ± 0.7	7.148 ± 2.382	19.26 ± 4.77
Sweroside	63.7 ± 17.6	2.1 ± 0.1	1.2 ± 0.3	261.2 ± 65.3	267.9 ± 70.8	3.9 ± 0.3	120.9 ± 36.95	785.1 ± 130.6

**Table 6:** Parent ion, daughter ion, DP, and CE of five analytes and IS in negative ion mode.

ID	Parent Ion (*m/z*)	Daughter Ion (*m/z*)	DP (V)	CE (eV)
Picroside-I	491.2	147.0	−75.20	−12.90
Picroside-II	511.3	234.8	−96.20	−33.30
Picroside-III	537.4	261.0	−101.29	−30.00
Minecoside	537.5	281.4	−84.92	−13.46
Sweroside	403.2	195.0	−45.86	−18.75
IS	525.2	449.1	−59.40	−20.02

## Data Availability

Not applicable.

## Sample Availability

Compounds Picroside-I, Picroside-II, Picroside-III, minecoside, sweroside and the *Picrorhiza scrophulariiflora* extraction are available from the authors.
